# Increased task-uncorrelated muscle activity in childhood dystonia

**DOI:** 10.1186/s12984-015-0045-1

**Published:** 2015-06-12

**Authors:** Francesca Lunardini, Serena Maggioni, Claudia Casellato, Matteo Bertucco, Alessandra L. G. Pedrocchi, Terence D. Sanger

**Affiliations:** Department of Electronics, Information and Bioengineering, NearLab, Politecnico di Milano, Via Giuseppe Colombo, 40, 20133 Milan, Italy; Department of Biomedical Engineering, University of Southern California, 1042 Downey Way, DRB 140, Los Angeles, CA 90089-1111 USA; Department of Child Neurology, University of Southern California, Los Angeles, CA 90089-1111 USA; Department of Biokinesiology & Physical Therapy, University of Southern California, Los Angeles, CA 90089-1111 USA; Children’s Hospital Los Angeles, Los Angeles, CA 90027 USA; Hocoma AG, Industriestrasse 4, CH-8604 Volketswil, Switzerland

**Keywords:** Childhood dystonia, Writing, EMG-kinematics, Spectral analysis, Task-uncorrelated activity

## Abstract

**Background:**

Even if movement abnormalities in dystonia are obvious on observation-based examinations, objective measures to characterize dystonia and to gain insights into its pathophysiology are still strongly needed. We hypothesize that motor abnormalities in childhood dystonia are partially due to the inability to suppress involuntary variable muscle activity irrelevant to the achievement of the desired motor task, resulting in the superposition of unwanted motion components on the desired movement. However, it is difficult to separate and quantify appropriate and inappropriate motor signals combined in the same muscle, especially during movement.

**Methods:**

We devise an innovative and practical method to objectively measure movement abnormalities during the performance of a continuous figure-eight writing task in 7 children with dystonia and 9 age-matched healthy controls. During the execution of a continuous writing task, muscle contractions should occur at frequencies that match the frequencies of the writing outcome. We compare the power spectra of kinematic trajectories and electromyographic signals of 8 upper limb muscles to separate muscle activity with the same frequency content of the figure-eight movement (task-correlated) from activity occurring at frequencies extraneous to the task (task-uncorrelated).

**Results:**

Children with dystonia present a greater magnitude of task-uncorrelated muscle components. The motor performance achieved by children with dystonia is characterized by an overall lower quality, with high spatial and temporal variability and an altered trade-off between speed and accuracy.

**Conclusions:**

Findings are consistent with the hypothesis that, in childhood dystonia, the ability to appropriately suppress variable and uncorrelated elements of movement is impaired. Here we present a proof-of-concept of a promising tool to characterize the phenomenology of movement disorders and to inform the design of neurorehabilitation therapies.

## Background

Childhood dystonia is defined as “a movement disorder in which involuntary sustained or intermittent muscle contractions cause twisting and repetitive movements, abnormal postures, or both” [1, 2]. Muscle activity in dystonia typically exhibits both overflow into task-unrelated muscles as well as greater variability within task-related muscles than in healthy subjects [3–5]. Increased variability may represent an inability to suppress motor noise [6], resulting in the superposition of unwanted motion components on the desired movement. This may lead to more variable and less efficient motor outcomes, which are typical in dystonia [7–9]. Even if movement abnormalities in dystonia are obvious on observation-based examinations, objective measures to characterize dystonia and to gain insights into its pathophysiology are still strongly needed.

During movement, it is difficult to separate voluntary from unwanted variable components of activity in muscles. Here, we apply a Fourier analysis to electromyographic (EMG) and kinematic data acquired during the performance of a continuous figure-eight writing task in children with dystonia and age-matched healthy controls. This new method exploits the frequency-domain features of cyclic motor tasks to discriminate between task-correlated and task-uncorrelated components within task-related muscles. We refer to “task-correlated” components to indicate muscle activity showing the same frequency content as the kinematics of the motor task. We define “task-uncorrelated” components as muscle components occurring at frequencies unrelated to the cyclic figure-eight movements, which include both fixed and variable elements that do not contribute to the achievement of the desired motor task.

We hypothesize that movement abnormalities in dystonia, reputedly related to basal ganglia dysfunction, are partially due to the inability to suppress involuntary variable activity unrelated to the task. Therefore, we expect children with dystonia to present significantly greater task-uncorrelated components of muscle activity compared to control subjects.

## Methods

### Participants

Inclusion criteria for this study were: I) primary or secondary dystonia; II) pediatric age (8-21 years); III) upper limb control impairment that does not prevent the writing task execution; IV) no cognitive impairment that prevents understanding of instructions; V) no Deep Brain Stimulation. Participants (Table 1) consisted of 7 children with dystonia (2 girls, 5 boys; ages 8–19 years, mean 12.6 ± 4.7 years) recruited from the Children’s Hospital Los Angeles and diagnosed by a pediatric neurologist, and a control group with 9 healthy children (7 girls, 2 boys; ages 12–20 years, mean 15.8 ± 4 years). The age distributions of the two groups were not statistically different. All participants with dystonia were rated on the Barry-Albright Dystonia (BAD) Scale [10] by two raters and the average score is reported in Table 1. The University of Southern California Institutional Review Board approved the study protocol. All parents gave informed written consent for participation and authorization for use of protected health information, and all children gave written assent. The study was performed in accordance with the Declaration of Helsinki.

**Table 1:** Characteristics of subjects

(A) Children with dystonia									
ID	Sex	Age	Diagnosis	Bad scale score				DOM. Arm	Medications
				R Arm	L Arm	Trunk	Total		
d1	M	106	Idiopathic primary generalized dystonia; DYT1-	1.5	1.5	0.5	5.5	R	Trihexyphenidyl (ARTANE), carbidopa-levodopa (SINEMET)
d2	M	234	Secondary generalized dystonia; mutation in TTPA (tocopherol transfer protein A) causing vitamin E deficiency	1.5	1.5	0.5	6	R	Vit E, botulinum toxin injections on sternocleidomastoid (L and R), levator scapulae (R), scalenus medius (R) 4 months before the experiment
d3	F	123	Secondary hemidystonia; traumatic brain injury	3	2	0.5	5.5	L	Trihexyphenidyl (ARTANE), Baclofen
d4	M	189	Primary segmental dystonia; DYT1-	1	1	0	2	L	Trihexyphenidyl (ARTANE)
d5	F	221	Secondary generalized dystonia; perinatal hypoxic ischemic injury	3	3	0.5	8.5	R	Trihexyphenidyl (ARTANE), carbidopa-levodopa (SINEMET)
d6	M	108	Secondary generalized dystonia; perinatal hypoxic ischemic injury	3	3	3	9*	L	Baclofen, botulinum toxin injections on Triceps (R), Biceps (R), and Flexor Carpi Ulnaris (R) 3 months before the experiment
d7	M	123	Secondary segmental dystonia; perinatal hypoxic ischemic injury	2.5	2.5	2	10	R	No medication
(B) Control children									
ID	Sex	Age						DOM. Arm	
c1	F	235						R	
c2	F	240						R	
c3	F	224						R	
c4	F	210						R	
c5	M	225						R	
c6	M	217						R	
c7	F	143						L	
c8	F	124						R	
c9	F	127						L	

A: Children with dystonia. Subject ID; Sex; Age (months); Diagnosis; Severity of Right (R) Arm, Left (L) Arm, Trunk, and Total Score (scores averaged over two raters are based on the Barry-Albright Dystonia Scale [10]; for each segment the score ranges from 0 - absence of dystonia - to 4 - severe dystonia); Dominant arm; Medications. [*Total Score NOT available]

B: Control children. Subject ID; Sex; Age (months); Dominant arm

### Apparatus

**Fig. 1:**
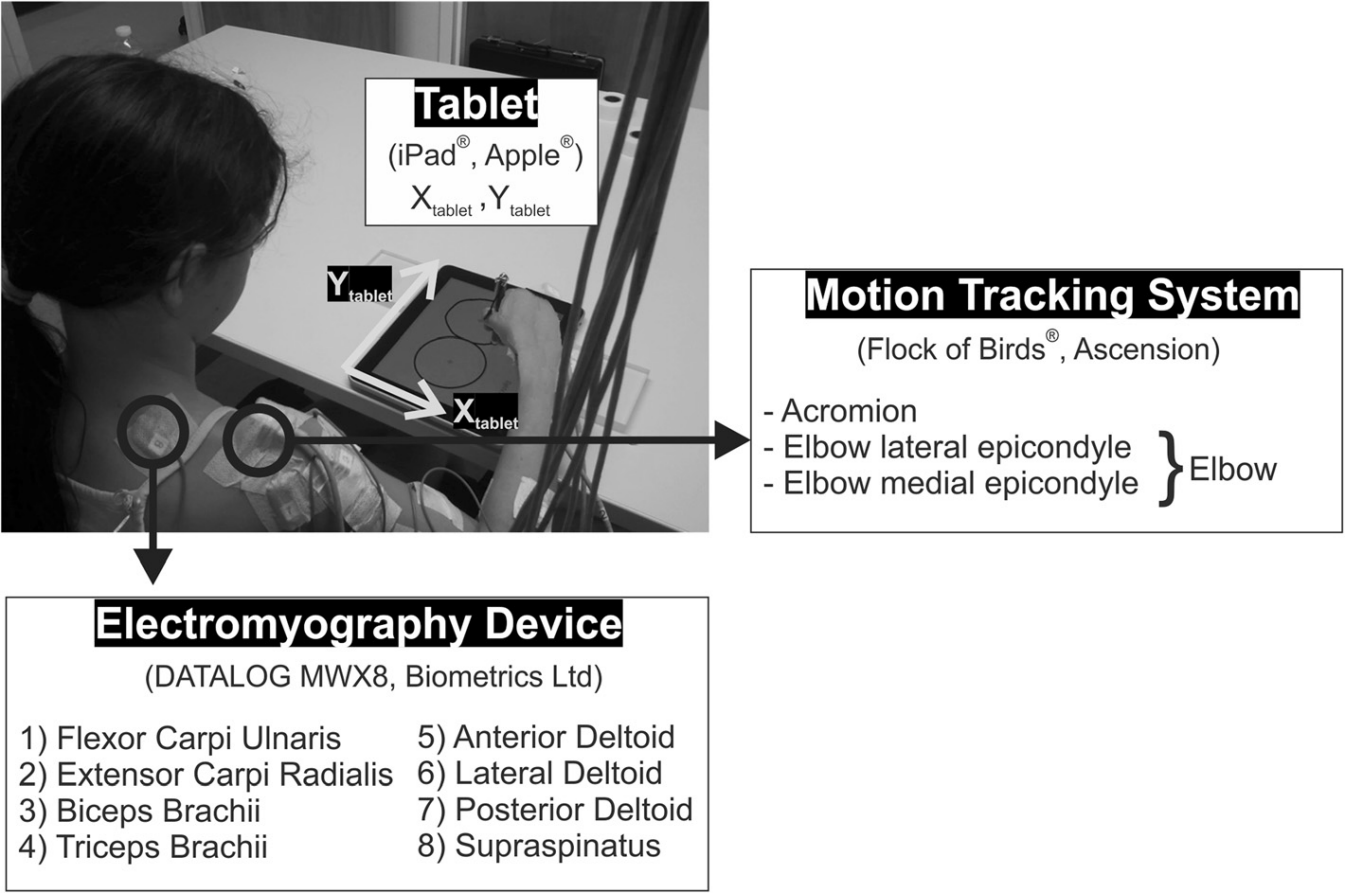
Setup. During the execution of the figure-eight writing task, subjects were seated at a height-adjustable desk. The apparatus included a motion tracking system (upper limb kinematics), an electromyography device (surface EMG of eight upper limb muscles), and a tablet (2D coordinates of the pen tip)

The acquisition system synchronized upper limb kinematics (Flock of Birds®, Ascension, Burlington, VT USA; 100 Hz sample frequency), EMG (DataLOG MWX8, Biometrics Ltd, Newport, UK; 1000 Hz sample frequency; 20-460 Hz bandwidth), and 2D coordinates of the pen tip on a tablet (iPad®, Apple®, Cupertino, CA USA; 60 Hz sample frequency). Kinematic sensors were placed on elbow lateral epicondyle, elbow medial epicondyle, and acromion of the upper-limb used to perform the task. Elbow coordinates were computed as the middle point between the two sensors on the elbow. For all the subjects, surface EMG signals were extracted from eight muscles of the upper limb that are known to contribute to wrist, elbow, and shoulder movements: Flexor Carpi Ulnaris (FCU), Extensor Carpi Radialis (ECR), Biceps Brachii (BIC), Triceps Brachii (TRIC), Anterior Deltoid (AD), Lateral Deltoid (LD), Posterior Deltoid (PD), and Supraspinatus (SS) (Table 2). Eight bipolar surface active electrodes (SX230 from Biometrics Ltd, Newport, UK), with an inter-electrode distance of 20 mm, were placed on the subjects. Prior to the placement, the skin over the muscles and the surface of the sensors were wiped with isopropyl alcohol pads to reduce electrical impedance at the skin electrode interface. The target muscles were mostly found by palpation, anatomical landmarks [11], and by visual inspection of the signal that gave the best response to clinical tests reported in Table 2. A single electrode was also placed on the subject’s contralateral wrist to serve as a ground and reference electrode. Prior to the start of the experiment, the EMG signals were displayed on a real-time monitor and visually inspected to ensure proper placement and quality of the signal. Custom software on the tablet was developed to record the 2D coordinates of the pen tip on the tablet (Cocos2d development environment; iOS 4.3 operating system; Apple®, Cupertino, CA USA). The subjects were seated on an armless chair, positioned at a distance from a height-adjustable table that allowed them to reach the furthest point of the tablet (fixed on the table in portrait orientation) with the elbow at 90 % of its maximum extension (Fig. 1). The subjects’ trunk was fastened to the seatback with a Velcro® belt to prevent the subjects from bending the trunk towards the table.

**Table 2:** Target muscles with related functions and clinical tests

Target muscles		Function	Clinical test
Flexor Carpi Ulnaris	FCU	Flexes and adducts the wrist, and may assist in flexion of the elbow	Flexion of the wrist toward the ulnar side, with the forearm in full supination and supported by the examiner
Extensor Carpi Radialis	ECR	Extends and abducts the wrist, and assists in flexion of the elbow	Extension of the wrist toward the radial side, with the forearm in slightly less than full pronation and rest on the table for support
Biceps Brachii	BIC	Flexes the shoulder joint and assists with shoulder adduction. Flexes the elbow and, with the origin fixed, supinates the forearm	Elbow flexion with the forearm in supination
Triceps Brachii	TRIC	Extends the elbow joint and assists in adduction and extension of the shoulder joint	Extension of the elbow joint, with the shoulder at 90° abduction, and with the arm supported by the table
Anterior Deltoid	AD	Flexes and, in the supine position, medially rotates the shoulder joint. Stabilizes the abduction of the shoulder joint.	Shoulder abduction in slight flexion, with the humerus in slight lateral rotation
Lateral Deltoid	LD	Abduction of shoulder joint	Shoulder abduction without rotation and with the elbow should be flexed
Posterior Deltoid	PD	Extends and, in the prone position, laterally rotates the shoulder joint. Stabilizes the abduction of the shoulder joint	Shoulder abduction in slight extension, with the humerus in slight medial rotation
Supraspinatus	SS	Abducts and laterally rotates the shoulder joint, and stabilizes the head of the humerus in the glenoid cavity during these movements	With the elbow bent, the arm is placed in abduction to shoulder level. Have the subject hold the position of slight anterior abduction and slight external rotation against pressure

### Protocols

Motor performance was studied during the execution of figure-eight writing movements. The subjects were asked to draw a figure-eight (15.7 (Y) cm × 7.8 (X) cm) on the tablet, following a displayed thin trace (0.3 cm thick). Prior to the start of the experiment, participants were encouraged to be as accurate as possible while tracing the figure-eight at their natural speed. Starting from the upper point of the figure-eight, subjects were requested to move in the mediolateral direction opposite to the arm used to perform the task. Subjects drew three sequences of ten figure-eight movements in a row. The task was performed with the dominant or preferred arm and subjects were asked not to lean their forearm on the table while writing.

### Data analyses

Data analyses were executed with Matlab® R2011a software (Mathworks®, Natick, MA USA). Statistical analysis was performed using RStudio® Version 0.98.981 (RStudio Inc.^©^, Boston, MA, USA).

#### Joint EMG-kinematic analysis

Before performing the spectral analysis, each EMG signal was processed with a band-pass Butterworth filter (5^th^ order, 5-400 Hz), and a stop-band Butterworth filter (5^th^ order, 60 Hz). A nonlinear recursive filter based on Bayesian estimation was applied. Compared to the traditional linear amplitude envelope, Bayesian filtering produces a smooth output that estimates the driving force underlying the EMG signal with low variability yet with the possibility of very rapid changes in output [12]. This filtering is essential to remove variability due to the surface EMG itself, so that all remaining variability is due to the neural control of muscle activity. Signals were then normalized to the maximum activation levels during movement, thus obtaining signals ranging from 0 to 1.

**Fig. 2:**
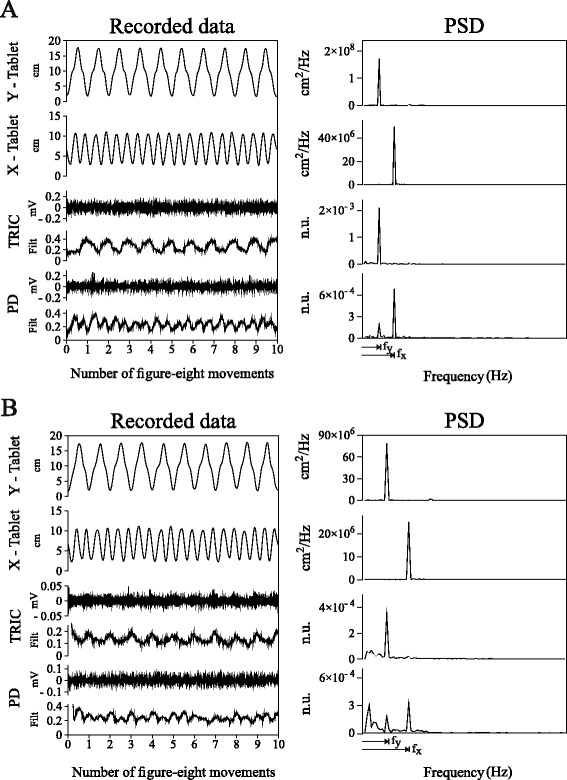
EMG-Kinematics Spectral Analysis. Panel **a**: Control subject (c2); Panel **b**: Subject with dystonia (d2). For each panel, from top to bottom: Tablet y-trajectory, Tablet x-trajectory, Triceps Brachii (TRIC) and Posterior Deltoid (PD) EMGs and non-linear envelopes (Filt) in a sequence of ten figure-eight movements represented in time (left column) and frequency (right column) domains. Note that Filt signals are normalized and dimensionless both in time and frequency domains (n.u.). f_y_ and f_x_ represent the subject-specific frequencies related to the vertical and horizontal components of the figure-eight

In order to detect the frequency features related to the motor outcome on the EMG signals, spectral analysis was applied to kinematic (Y_tablet_ and X_tablet_) and EMG data (normalized Bayesian filtering outputs). Each kinematic and EMG signal was pre-processed as follows: I) the sequence was divided into the 10 single figure-eight movements; II) each kinematic and EMG movement was re-sampled to equalize the duration of the figure-eight movements between all subjects; III) re-sampled movements were re-assembled in order to rebuild the sequence; IV) the signal was linearly de-trended; V) Fourier Transform (FT) of the re-sampled sequence was computed. We then computed the Power Spectral Density (PSD) based on the FT coefficients for kinematic and EMG signals. In the figure-eight, the horizontal (f_x_) and vertical (f_y_) frequency components are expected to be in a ratio of 2:1 (f_x_ = 2 * f_y_). As a result, for each subject, the Y_tablet_ PSD presented a well-defined peak at the frequency related to the mean duration of the figure-eight movement (f_y_), while the X_tablet_ PSD showed a peak at double the figure-eight frequency (f_x_). Spectral peaks at exactly the same frequencies, f_y_ and f_x_, were clearly detectable also in the EMG PSDs (Fig. 2). The sum of the spectral energy of the peaks at f_y_ and f_x_ (*P*_*y*_ *+ P*_*x*_) was regarded as an index of muscle activity that contains the frequency components of the kinematic task, and it was computed for each muscle individually. All PSD components at frequencies other than f_y_ and f_x_ were considered task-uncorrelated, representing noisy and variable components that do not contribute to the desired cyclical task, together with corrective activity to counter possible errors generated while non-efficiently tracing the figure-eight. The more noise that characterizes the EMGs, the more we expected the task-uncorrelated components to increase compared with the task-correlated components. The ratio between *P*_*y*_ *+ P*_*x*_ and the full spectrum energy ranging from 0 to 5 Hz was calculated for each muscle as an indication of the relative contribution of the task-correlated components (*task-correlation index*) (Table 3). For each subject, for each muscle, the *task-correlation index* was computed for each sequence of ten single figure-eight movements and averaged over the three sequences performed.

**Table 3:** Task-correlation index

(A) Children with dystonia								
ID	*Task-correlation index*							
	FCU	ECR	BIC	TRIC	AD	LD	PD	SS
d1	0.264	0.230	0.277	0.414	0.622	0.222	0.336	0.067
d2	0.258	0.133	0.674	0.592	0.668	0.153	0.419	0.330
d3	0.124	0.255	0.238	0.429	0.481	0.432	0.524	0.150
d4	0.370	0.732	0.390	0.206	0.534	0.156	0.364	0.072
d5	0.453	0.246	0.486	0.418	0.586	0.324	0.453	0.218
d6	0.273	0.140	0.244	0.279	0.658	0.382	0.318	0.219
d7	0.454	0.117	0.245	0.409	0.256	0.130	0.333	0.236
(B) Control children								
ID	*Task-correlation index*							
	FCU	ECR	BIC	TRIC	AD	LD	PD	SS
c1	0.152	0.157	0.776	0.816	0.558	0.063	0.526	0.464
c2	0.602	0.196	0.385	0.692	0.810	0.724	0.684	0.436
c3	0.485	0.602	0.581	0.586	0.711	0.514	0.291	0.432
c4	0.414	0.029	0.645	0.058	0.291	0.185	0.504	0.240
c5	0.077	0.414	0.375	0.345	0.656	0.243	0.688	0.527
c6	0.763	0.441	0.740	0.075	0.715	0.396	0.556	0.309
c7	0.237	0.518	0.291	0.489	0.651	0.605	0.649	0.418
c8	0.066	0.232	0.545	0.319	0.373	0.099	0.416	0.156
c9	0.200	0.574	0.605	0.545	0.691	0.275	0.475	0.120

A: Children with dystonia; B: Control children: Subject ID and task-correlation index for all eight muscles: Flexor Carpi Ulnaris (FCU), Extensor Carpi Radialis (ECR), Biceps Brachii (BIC), Triceps Brachii (TRIC), Anterior Deltoid (AD), Lateral Deltoid (LD), Posterior Deltoid (PD), and Supraspinatus (SS)

#### Kinematic analysis

Kinematic data were processed with a low-pass Butterworth filter (5^th^ order, 3 Hz). The cutoff frequency was determined by the “Jackson Knee” method [13].

The *accuracy error* of the figure-eight trace on the tablet was computed as the root mean square error between the actual pen trajectory and the displayed figure-eight trace for each movement separately, and the value was then averaged over all movements. The *speed* was computed for each figure-eight movement and then averaged over the thirty single movements (three sequences of ten movements). Moving fast and accurately can be considered a central goal of the motor performance. Since motor speed and accuracy interact, to quantify motor performance, it is important to measure them together [7, 14–16]. Therefore, we examined motor skill during the writing task by computing the ratio between accuracy error and speed (*AccErr/Speed*) for each movement separately and we averaged it over the thirty movements. The intra-subject *spatial variability* was estimated as the average standard deviation of the trajectories after time alignment. As proposed in [17], time alignment was achieved by phase shifting the data in the frequency domain. In particular, after re-sampling all the figure-eight movements separately to equalize their duration, the FT of the module of the position vector of each figure-eight movement was computed and all the FT components of the *i-th* figure-eight movement were shifted by ϴ(f_yi_), where ϴ(f_yi_) is the phase of the frequency component related to the duration of the *i-th* figure-eight movement (f_yi_). The signals were then reconstructed by applying an inverse FT. As a result, the different movements were aligned in time and the variability was determined only by spatial characteristics. *Spatial variability* was evaluated for tablet, elbow, and acromion trajectories. The intra-subject *temporal variability* was quantified as the standard deviation among the durations of each movement.

#### Statistical analysis

Due to the small sample size, we ran nonparametric statistics. Nonparametric aligned rank test for interaction in two-way factorial designs with repeated measures (R-package ‘npIntFactRep’) was applied to investigate a possible between-group difference in the *task-correlation index*. The model included a between factor with 2 levels (Group) and a within factor with 8 levels (Muscle). For the kinematic parameters, to look for possible between-group differences, we used the nonparametric Kruskal-Wallis rank sum test. Spearman’s rank correlation coefficient was used to investigate the presence of statistical dependence between the *task-correlation index* and all the kinematic parameters separately. For children with dystonia, the Spearman’s rank correlation coefficient was also studied between the *task-correlation index* and the BAD score of the arm used to perform the task (BAD_arm_), and the BAD total score (BAD_total_) (Table 1). For all tests, the significance level was set at 5 %.

## Results

Results are presented as medians and lower and upper quartiles.

### EMG results

**Fig. 3:**
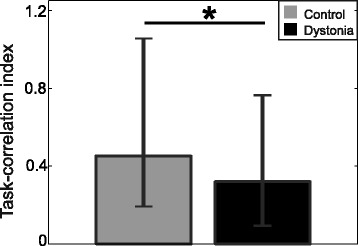
Between-group difference in the task-correlation index. Medians (bars) and lower and upper quartiles (whiskers) of the task-correlation index over all eight muscles for control children (gray) and children with dystonia (black). The task-correlation index is significantly lower (*) in children with dystonia, compared to controls

Significant effects was found for the independent factor Group [*p = 0.049*], with no significant effect of the within-subject factor Muscle and no significant interaction. The *task-correlation index* was higher for controls [Control: 0.453 (0.259-0.603); Dystonia: 0.321 (0.226-0.442)], indicating that, for healthy children, 45 % of muscle activity was correlated with the task, whereas only 32 % was correlated for children with dystonia (Fig. 3). The finding supported the prediction of a greater magnitude of task-uncorrelated components in the EMGs of subjects with dystonia.

### Kinematic results

**Fig. 4:**
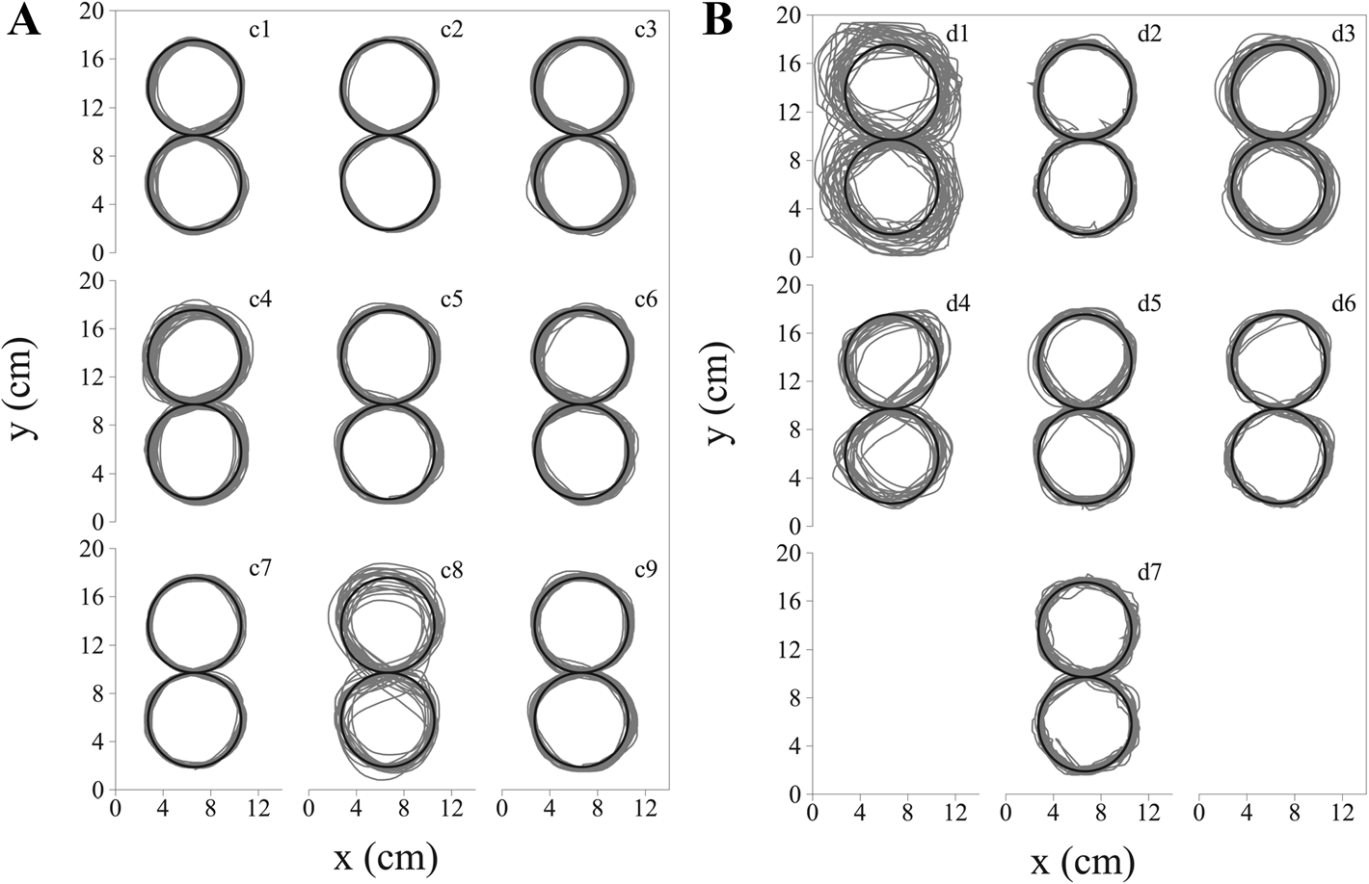
Figure eight writing outcome on the Tablet. Panel **a**: Control children; Panel **b**: Children with dystonia. For each subject the superposition of all 30 figure-eight drawings on the Tablet is presented (gray). The black line is the figure-eight trace provided

No significant difference between the two groups was found either for the *accuracy error* [Control: 0.255 (0.234-0.298); Dystonia: 0.335 (0.282-0.563) cm], or for the *speed* [Control: 18.341 (11.282-20.382) cm/s; Dystonia: 10.351 (6.101-14.187) cm/s] of the final outcome. A large range of movement speeds was reported for both children with dystonia [6.075-27.892 cm/s] and age-matched control children [8.071-33.040 cm/s]. This is not surprising since the task did not impose any time constraint, while participants were asked to pay particular attention to accuracy while following the trace provided. A difference between the two groups was found when examining *AccErr/Speed*, which was significantly increased in children with dystonia after controlling for the effect of age [Control: 0.017 (0.014-0.019) cm/(cm/s); Dystonia: 0.034 (0.026-0.051) cm/(cm/s), *p = 0.002*]. At the same execution speed, children with dystonia performed, on average, a movement leading to a less accurate outcome, meaning that the speed-accuracy trade-off is altered in dystonia. *Spatial variability* was significantly increased in children with dystonia for tablet, elbow, and acromion trajectories [Tablet: Control: 0.3612 (0.354-0.433) cm; Dystonia: 0.806 (0.670-0.876) cm, *p = 0.001;* Elbow: Control: 0.101 (0.096-0.123) cm; Dystonia: 0.333 (0.178-0.575) cm, *p = 0.003*; Acromion: Control: 0.317 (0.262-0.358) cm; Dystonia: 0.728 (0703-1.127) cm, *p = 0.003*]. Children with dystonia presented significantly increased *temporal variability* compared to healthy controls [Control: 0.352 (0.328-0.756) s; Dystonia: 1.603 (1.125-2.739) s, *p = 0.030*]. Overall, the motor performance of children with dystonia was characterized by increased variability both in temporal and spatial terms (Fig. 4). When investigating statistical dependence between the *task-correlation index* and the computed kinematic parameters, significant results emerged for *accuracy error* [rho (ρ) = -0738; *p = 0.001*] and *spatial variability* of tablet [ρ = -0621; *p = 0.012*]. The negative ρ values show that the *task-correlation index* decreases when spatial variability and inaccuracy of the end-effector increase. No significant linear regression was reported between the *task-correlation index* and either the BAD_arm_ or the BAD_total_ scores for children with dystonia. The lack of correlation between the *task-correlation index* and the BAD Scale score may be due to the small sample size (7 children with dystonia) and to the limited range of the BAD Scale (4-point scale for each segment). However, it is likely that the measure here proposed captures a different type of movement abnormality in dystonia, mostly task-dependent, as supported by the correlation with the kinematic parameters.

## Discussion

Our results show that, compared to age-matched healthy controls, the motor performance of children with dystonia is characterized by increased task-uncorrelated muscle components. This finding is consistent with the hypothesis that, in dystonia, the ability to appropriately suppress variable and uncorrelated muscle activity is impaired. The method here presented devises an innovative approach to quantify abnormal sub-components of muscle activity by applying a simple and well-known analysis to movement outcome and relative muscular control signals. The comparison of these task-uncorrelated muscle components between children with dystonia and healthy subjects allows us to gain insights into the underlying mechanisms responsible for the clinical phenomenology of dystonia. The proposed method is rather conservative, indeed it may underestimate the abnormalities in dystonia because it ignores involuntary activity that occurs at task-relevant frequencies. Muscle activity may reflect a combination of i) voluntary activity of muscles needed to accomplish the task, ii) involuntary overflow of activity into other muscles, iii) involuntary variable activity uncorrelated to the task. Our method extracts and quantifies the latter, while it is not designed to measure overflow of voluntary activity from task-relevant muscles, since voluntary activity is task-correlated. Nevertheless, this method provides a promising new technique for identifying one component of abnormal activity in specific muscles during movement, and it documents superimposed task-uncorrelated patterns of activity in dystonia.

The effectiveness of the proposed method as a measure to quantify abnormalities in dystonia is further validated by the significant linear correlation with kinematic parameters such as the spatial variability and the accuracy error of the final outcome. The task-correlation index addresses the need for a quantitative and objective assessment of movement abnormalities in dystonia during the execution of specific motor tasks. On the other hand, the BAD Scale was developed to assess, through observation-based examinations, varied functional limitations due to dystonia in persons with Cerebral Palsy and Traumatic Brain Injury, and it is not related to any specific task [10]. Thus, the measure we propose aims at integrating the BAD Scale severity score, rather than replacing it.

Results show that the motor performance achieved by children with dystonia is characterized by an overall lower quality, with high variability and an altered trade-off between speed and accuracy. Children with dystonia exhibited increased spatial variability at all levels of the upper body. Indeed, tablet, elbow, and acromion trajectories were less repeatable across trials. In addition, the analysis of temporal variability revealed a behavior significantly less consistent in time for the group with dystonia. The finding is consistent with a previous study from our team [6], that reported evidence of increased motor variability in dystonia due to the inability to remove noisy components. When analyzing the speed of movement, a wide range of speeds was found across the subjects of both groups, and no significant difference between children with and without dystonia was reported. The result is not surprising, since the task did not impose any time constraint. However, a significant difference between the two populations emerged in the ratio between accuracy error and speed of movement, which was higher for subjects with dystonia. In agreement with previous studies [7, 15], the result shows that the nature of the relationship between speed and accuracy is altered in dystonia. Since the signal-dependent noise that affects the sensorimotor system inherently imposes a trade-off between speed and accuracy of movement [18], the observed altered trade-off supports the hypothesis of increased signal-dependent noise in dystonia [15]. In other words, optimal control theories [19] assert that, when performing upper-limb tasks, subjects minimize the variance of the arm position for a specified duration or the movement duration for a specified positional variance. This criterion is met in a less effective way in dystonia, since the neural control signals are corrupted by a noise whose variance is more sensitive to the size of the signals themselves than in healthy systems [15, 20].

Both primary and secondary dystonia are associated with dysfunction or injury to the basal ganglia [4, 21, 22]. Neurophysiological studies support the theory that, when voluntary movement is generated, the basal ganglia are responsible for the focused selection of the desired motor pattern and for the inhibition of undesired and competing movements [23–25]. Our finding is consistent with the hypothesis that abnormalities arising from the basal ganglia in dystonia may be related to the inability to remove unwanted potential movements or elements of movement. As a consequence, movement in childhood dystonia may represent the superposition of unwanted and variable motion components on the desired movement. Nevertheless, it is worth noting that, in many cases, the injury is not limited to basal ganglia, and damages to other brain areas like cerebellum, brainstem, or sensory cortex can be the cause of dystonia. In addition, the uncorrelated muscle components detected during the execution of the figure-eight task may capture activity other than involuntary muscle noise arising from underlying central motor commands problem. For instance, the increased task-uncorrelated components found in dystonia may also partially consist of activity reflecting non-efficiency of movement, such as corrective activity to counter possible errors generated while tracing the figure-eight, or activity due to variations in grip postures. For these reasons, the nature and the neural substrate related to these unwanted noisy elements would need further investigations, perhaps involving functional imaging.

The main weakness of the current study is represented by the small sample size. To increase the robustness and the generalizability of our results, further assessment of the value of these measurement techniques will require a larger sample of subjects, with a balance between boys and girls in each group. Such future studies should unravel the issue of whether the speed-accuracy trade-off may be influenced by the difference in the composition of the two groups, since it is known that boys tend to exhibit increased variability of movement [26]. Moreover, a larger sample size would allow us to take into account the effect of age as a covariate and to further investigate the relationship between our outcome measures and the BAD Scale score. Finally, a larger sample of subjects may be recruited to test the reliability and the sensitivity of the proposed index as a tool that may be used in clinical practice to detect, at the individual level, increased magnitude of task-uncorrelated components of muscle activity.

## Conclusion

The present work represents a proof-of-concept of a potentially useful new method to objectively measure movement abnormalities in childhood dystonia in terms of deviations from normal motor control principles, thus offering the possibility to characterize the phenomenology of dystonia and gaining insights into its pathophysiology. Our simple and innovative approach applies spectral analysis to motor outcomes and related muscle patterns during the performance of a continuous figure-eight writing task to easily distinguish muscle activity correlated with the task from uncorrelated involuntary components.

The results reported here show that, compared to age-matched healthy controls, children with dystonia present increased task-uncorrelated components in multiple muscles, and increased spatial and temporal variability. These findings are consistent with the hypothesis that, in dystonia, the ability to appropriately suppress unwanted and irrelevant muscle activity is impaired, resulting in the superposition of unwanted motion components on the desired movement. As a consequence, we observed more variable and less efficient motor outcomes in children with dystonia compared to age-matched healthy controls.

The investigative tool presented here has the potential to inform the design of neurorehabilitation therapies for dystonia. The study’s finding fits with the idea that dystonia is a central motor control problem, in which appropriate and inappropriate motor signals can combine in the same muscle. Such a finding helps to speculate on the mechanisms underlying the phenomenology of dystonia and, as a consequence, may be useful to drive the design of novel techniques that will intervene by modifying central signals, such as neural prostheses, Deep Brain Stimulation, Transcranial Magnetic Stimulation, or Peripheral Nerve Stimulation intended to selectively modify central connections.
